# Soil Sealing and Hydrological Changes during the Development of the University Campus of Elche (Spain)

**DOI:** 10.3390/ijerph18189511

**Published:** 2021-09-09

**Authors:** Manon Navarro-Leblond, Ignacio Meléndez-Pastor, Jose Navarro-Pedreño, Ignacio Gómez Lucas

**Affiliations:** Department of Agrochemistry and Environment, University Miguel Hernández of Elche, Avenida de la Universidad de Elche s/n, Edificio Alcudia, 03202 Elche, Spain; manon.navarro@alu.umh.es (M.N.-L.); imelendez@umh.es (I.M.-P.); ignacio.gomez@umh.es (I.G.L.)

**Keywords:** green infrastructure, irrigation system, land changes, soil sealing, urbanization

## Abstract

The University Miguel Hernández of Elche was created in 1996 and its headquarters is located in the city of Elche. A new campus was developed where new buildings and infrastructures have been established for over 25 years in the north of the city. The university is growing, and the land cover/land use is changing, adapted to the new infrastructures. In fact, the landscape changed from a periurban agricultural area mixed with other activities into an urbanized area integrated into the city. The purpose of this work was to evaluate the progressive sealing of the soil and the consequences on the surface hydrology. The area is close to the Palmeral of Elche, a landscape of date palm groves with an ancient irrigation system, which is a World Heritage Cultural Landscape recognized by UNESCO. The evolution of the land occupation was analyzed based on the Aerial National Orthophotography Plan (PNOA). Soil sealing and the modifications of the hydrological ancient irrigation system were detected. Based on the results, proposals for improvement are made in order to implement green infrastructures and landscape recovery that can alleviate the possible negative effects of the soil sealing in the area occupied by the university.

## 1. Introduction

Soil formation is considered a very slow process that results in a complex and dynamic system with continuously changing properties [1]. Soil acts as a producer, filter-buffer, carrier, resource, habitat, and cultural heritage, and a number of crucial functions have been recognized to be environmentally, economically, and socially important [2,3,4,5]. However, since the emergence of the first settlements the soil has been widely transformed and degraded by anthropogenic activities [6]. According to the FAO’s key results [7], globally, about 33% of the land surface is already degraded and over 90% could become degraded by 2050.

Land changes reflect socioeconomic development and political decisions across time, becoming a relevant factor in understanding the dynamics of the relation between soil functions and land use/land cover (LULC). It is commonly accepted to define land use as urban landscapes (e.g., industrial, residential areas, etc.) that link land cover to human activities, and land cover as land-use components (e.g., vegetation, concrete, etc.) that represent the biophysical conditions of the earth [8]. Over the past century, several factors, such as better living standards, greater disposable incomes, or climatic amenities, have driven to rapid land-cover changes along Euro-Mediterranean coastal areas [9,10]. In the Valencian Community, agricultural landscapes have been extremely modified by urbanization processes and soil degradation has been identified as the main threat [11,12]. Land degradation implies a progressive loss of soil functionality that diminishes its capacity to provide goods and services, including biological, hydrological, social, and economic services [13]. In this context, soil sealing is the most intense form of land-take or land consumption and can be defined as the permanent covering of land by completely or partially impermeable artificial material (e.g., asphalt, concrete, metal, glass, and plastic) [3,14].

As soil sealing is related to a change in land cover and its associated properties over time [15], the exchange and transformation of the energy and matter of each land-cover unit can be analyzed [16] (e.g., urban climate, hydrology, energy demands for space heating, and carbon dioxide emissions). The extent of impervious surfaces involves severe consequences to the biological, hydrological, and atmospheric compartments [17], for instance, the (i) loss of infiltration and baseflow; (ii) increase in runoff rates, volumes, and response to rainfall; (iii) air and water pollution; (iv) habitat fragmentation and loss of biodiversity; (v) greenhouse gas emissions through interruption of carbon sink; and (vi) the urban heat island effect from the decrease in radiation absorption [2,17,18,19,20]. In addition, soil sealing may have a great impact on neighboring ecosystems by changing water pathways and exposing unsealed soils to pollution [2]. Nowadays, the negative effects of urban sprawl are fully recognized by the European Union, where, on the average, around 9% of the surface is covered with impermeable material [21].

Extensive work related to the change in runoff characteristics has been carried out at urban scales ranging from water quality research [8,22,23] to runoff spatial–temporal variations [19,24,25]. For example, in the impervious surface of a roundabout located on the campus of the Polytechnic University of Valencia, the results of Andrés-Doménech et al. [26] showed high event mean concentrations (EMCs) for suspended solids, organic matter (COD, BOD), nutrients, and metals under Mediterranean climate conditions, exceeding, on average, the maximum acceptable value fixed in the European Directive 91/271/ECC for TSS and the environmental quality standards (EQS) set out in the European Directive 2008/105/CE for Cu, Ni and Pb. Pollutant first-flush, considered as “the disproportionate discharge of either higher pollutant concentrations or load in the initial part of a runoff event relative to its latter part” [23], has been identified as an important phenomenon that causes detrimental impacts on the quality of receiving bodies [27]. With climate change predicted to increase rainfall intensities and extreme events [28,29], water body quality preservation must be a priority, especially in semiarid areas where water availability is already scarce. In addition, a deep understanding of hydrological behavior in urban Mediterranean environments is necessary to develop planning strategies that improve resilience to these events.

To better represent the temporal and spatial variability of small-scale rainfall–runoff processes, detailed temporal and spatial information on land cover and hydrological parameters are required [8]. Land-cover classification provides input variables for a wide variety of environmental models (e.g., land change, habitat and biodiversity, ecosystem services, earth trends, climate change adaptation, hydrological response, etc.) [15,30]. With the computational capabilities and the availability and accessibility of digital spatial data, the Geographic Information System (GIS) and remote sensing (RS) have become versatile tools in decision support systems, which can be applied in a combination of hydrological models to analyze the impacts of urbanization on flood behavior [15]. However, a large number of input parameters are necessary to measure and predict rainfall excess with high levels of precision in watershed models such as the Storm Water Management Model (SWMM) or the Hydrologic Modeling System developed by the Hydrologic Engineering Center (HEC-HMS) [31].

Consequently, empirical methods are more effective for estimating runoff volumes when data are not available or under semiarid and Mediterranean environments [24]. The most common example of this type of approach is probably the Soil Conservation Service Curve Number (SCS-CN) method, which has been applied worldwide to assess the effects of land cover change on surface runoff [32,33], given its automaticity and simplicity. In Spain, it was adapted by Témez [34] and constitutes the basis of the Standard 5.2-IC of road surface drainage.

In the last two decades, an increasing number of universities have started to improve their own sustainability strategies in order to reduce their campuses’ environmental impact [35]. Amaral et al. [36] carried out a review of the implemented actions and initiatives on university campuses as reported in scientific publications, showing that the largest initiatives were aimed at building improvements, while only 6% of the cases included hydrological projects (wastewater treatment and rainwater harvesting for irrigation of green spaces). Against this backdrop, this paper contributes to the literature by exploring the impacts of soil sealing on surface runoff on the headquarter campus of the University Miguel Hernández of Elche (UMH), by accounting for land-cover changes throughout its development (1997–2017). To this end, the surface cover classification was carried out from manual photointerpretation of high-resolution aerial images. Then, two periods were analyzed (1997–2007 and 2007–2017) to assess land-cover changes. Finally, runoff production was estimated under three development scenarios for the university campus of Elche, expecting to set a precedent for developing integrated water management practices that allow rainwater collection, treatment, storage, and reuse, for example, by using sustainable and holistic approaches like Best Management Practices (BMPs), Low Impact Developments (LIDs) or Sustainable Urban Drainage Systems (SUDS).

## 2. Materials and Methods

### 2.1. The Study Aarea

**Figure 1:**
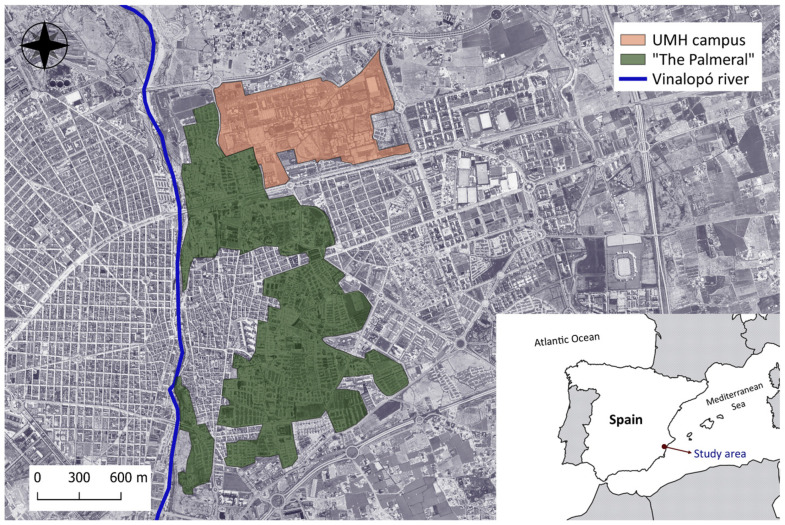
Study area location.

The UMH campus is located in SE Spain, in the Mediterranean city of Elche (Figure 1). The study area extends over 68.8 ha and rises at an altitude range of 88–100 m close to a historical agricultural landscape of great cultural value included in the UNESCO-World Heritage List since 2000 (nº 930): the Palmeral of Elche. This is a landscape of groves of date palms associated with an old agricultural irrigation system, historically documented since at least the 10th century AD and considered a remarkable example of sustainable water management [37]. Four ancient irrigation channels cross in the campus area used to provide water for several irrigated parts of the municipality.

Elche is characterized by a semi-arid Mediterranean climate with mild winters and dry and hot summers. The average annual precipitation and mean temperature are around 289 mm and 17.6 °C, respectively [38]. Additionally, three reasons make this region extremely vulnerable [39,40]: (1) Water demand in semi-arid areas cannot be fully supplied by conventional water resources, even considering wastewater reuse and desalination; (2) Total annual precipitation has been reduced by up to 15% in the last three decades in Alicante, and water availability is expected to continue to decline by 2050, which increases pressure on water resources; and (3) Rainfall occurs mainly in autumn, when intense rainfall and flash floods are more frequent. Consequently, it may cause considerable damage and incur great costs (e.g., September 2019 in Vega Baja del Segura, region south of the province of Alicante).

Considering the projection of drier conditions and under the uncertainty of high rainfall event occurrences in the future [39,41], the campus of Elche became a suitable case study to assess on a small scale the impact of land-cover changes on surface runoff and to propose conservation measures to move toward greater water self-sufficiency and reduce soil sealing.

### 2.2. Land-Cover Mapping

In this work, a time series of aerial images compiled between 1997 and 2017 was used to create a database of chronological land cover maps, of which three specific years were used to assess the dynamics of land cover on the campus: 1997, 2007, and 2017. Cartographic data required for the mapping of the study area was obtained from the National Geographic Institute (IGN) [42], where two types of images were available: first, aerial photos from the Five-Year flight 1998–2003, from which year 1999 was selected. The spatial resolution for this image was 1 m. Second, orthophotos were selected from the olive cultivation (GIS-OLISTAT), the Geographic Information System of Agricultural Parcels (SIGPAC), and the National Plan of Aerial Photography (PNOA). Orthophotos were obtained for the years 1997, 2002, 2005, 2007, 2009, 2012, 2014, and 2017 at spatial resolutions of 1, 0.5, 0.5, 0.5, 0.25, 0.5, 0.25, and 0.25 m, respectively. Georeferencing was carried out for the year 1999 by using the free and open-source geographic information system QGIS [43]. Also, official cartography related to the campus boundaries and hydraulic infrastructure was provided by the UMH Infrastructure Service.

Each aerial photograph was digitized by visual interpretation using a land-cover classification subjectively decided. Additionally, in the last year (2017), a field validation survey was accomplished. Surface classes established are shown in Table 1, where bitumen, cement, and concrete surfaces, stabilized earth roads, artificial grass, and mixed land covers were defined as soil sealed. Several nomenclatures have been reported in urban scales. For example, Rio et al. [8] used two levels for land-cover classification in terms of water quantity and quality modeling (Level 1: Hydrological Response Units; Level 2: Water Quality Response Units), while in Zhao et al. [44], categories were defined to assess their thermal contributions.

Although many different vegetation classifications are identified in rural catchments, in this case only crops and density of vegetation in urban green areas were considered to estimate the initial abstraction in the rainfall–runoff process, which may include trees, shrubs, and grass.

Land-cover change detection was performed with the TerrSet software developed by ClarkLabs [30]. For this purpose, digitized polygons were converted to raster format and two periods were analyzed: 1997–2007 and 2007–2017. As a result, each cross-tabulation matrix was examined to assess the net change, persistence, swap, gain, and loss of land categories between time 1 and time 2 following the Pontius et al. [45] approach. Categories from time 1 and time 2 configure the rows (*P_i_*) and the columns (*P_j_*), respectively, of the transition matrix, which ends with an additional column (*P*_*i*+_) and row (*P*_+*j*_) to denote the total surface for each category in time 1 and time 2. The diagonal entries indicate the total amount of surface that remained constant during the time interval considered, while the rest of the cells reflect the surface that changed to another category.

Gain (*G_j_*) Equation (1), loss (*L_i__j_*) Equation (2), net change (*C_n_*) Equation (3), and swap (*S_j_*) Equation (4), can be calculated as follows [45]:

(1) $$G_{j}=\left(P_{+j}\right)-\left(P_{jj}\right),$$

(2) $$L_{ij}=\left(P_{i+}\right)-\left(P_{jj}\right),$$

(3) $$C_{n}=\left|P_{+j}-P_{i+}\right|,$$

(4) $$S_{j}=2\cdot MIN\;\left(L_{ij},G_{j}\right)$$

where *P*_+*j*_ represents the total area of category *j* in time 2; *P_i_*_+_ is the total area of category *i* in time 1, and *P_jj_* denotes the persistence.

### 2.3. Surface Runoff Estimation

#### 2.3.1. The SCS-CN Method

Surface runoff was estimated using the empirical Soil Conservation Service Curve Number (SCS-CN) method, which was developed in 1972 by the Soil Conservation Service (SCS) and recently integrated into several hydrological models, such as SWMM, HEC-HMS, or the Soil and Water Assessment Tool (SWAT). Two parameters are mainly considered in this method: precipitation (*P*) and initial abstraction (*I_a_* or *P*_0_). The initial abstraction is assumed to be a function of the potential maximum soil moisture retention (*S*) and represents the threshold from which runoff begins. Estimating *P*_0_ is not easy, however it can be expressed as (Equation (5)):

*P*_0_ = *λ*·*S* (5)

where *λ* denotes the initial abstraction ratio that is assumed to be constant (*λ* = 0.2 in this study).

There is a dimensionless characteristic numbered curve (CN) related to each soil-cover complex ranging from 0 to 100 that reflects its hydrological behavior and runoff potential. Equation (6) shows the transformation of CN to *S* (in mm):

(6) $$S=\frac{25,400}{CN}-254$$

Tabulated *P*_0_ and CN values are provided by the Spanish Standard 5.2-IC of surface drainage [46] and the National Engineering Handbook Hydrology (NEH) of the NRCS [47], respectively. *P*_0_ and CN equivalence is shown in Equation (7).

(7) $$P_{0}=\frac{5080}{CN}-50.8.$$

Then, direct runoff (channel runoff, surface runoff, and subsurface flow in unknown proportions) for a given precipitation event and assuming *λ* = 0.2 can be calculated as fo-llows (Equation (8)):

(8) $$\begin{array}{cc} E=0 & \mathrm{for}\;P\leq P_{0}\\ E=\frac{{\left(P-P_{0}\right)}^{2}}{\left(P+4P_{0}\right)} & \mathrm{for}\;P>P_{0} \end{array}$$

where *E* denotes the depth of runoff in mm, *P* represents the depth of rainfall in mm and *P*_0_ is the initial abstraction in mm. Lower *P*_0_ values (or higher CN values) indicate that the surface has a higher potential for runoff production.

Both, *P*_0_ and CN, are mainly determined by land cover type, slope, degree of previous soil moisture (AMC), and hydrologic soil group (HSG). According to the infiltration capacities of soils, four HSGs (A, B, C, and D) are distinguished, from sandier (A) to more clayey textures (D). Furthermore, three types of AMC are defined in the SCS-CN method: I (dry conditions), II (average conditions), and III (wetter conditions).

#### 2.3.2. Initial *P*_0_, HSG, and Weighted *P*_0_ in the Area Study

The allocation of HSGs remains one of the major uncertainties of this method [48], especially in semi-arid areas. Thus, in the present study, the proposal of Camarasa et al. [24], which considers lithological, geomorphological, and soil factors, was selected and compared with the HSG map derived from the Standard 5.2-IC of surface drainage. As a result, HSG C was assumed for the campus area, which is sited on alluvial plains of Quaternary sediments (gravels, sands, and clayey silts) [49] and is characterized by the presence of haplocalcids [50].

Initial *P*_0_ and CN values for each land cover classification are shown in Table 2. Ta-bulated *P*_0_ values were selected for an average slope of the campus less of than 3%, which was calculated from a digital elevation model with a cell size of 5 m [42], and the transformation of CN into *P*_0_ was carried out with Equation (7). These values assume an AMC II that can be considered standard when the Procedure is not applied to real rainfall events [51], as in the current case. However, the Standard 5.2-IC provides a corrector coefficient based on the area location and return period that will be applied.

For a watershed having more than one hydrologic soil-cover complex, a weighted *P*_0_ value can be estimated if the area of each land cover is known [51]:

(9) $$P_{0}^{i}\left(w\right)=\underset{i}{\sum }S_{i}\cdot \;P_{0}\left(i\right)$$

where *P^i^*_0_ (*w*) is the initial weighted *P*_0_; *S_i_* is the fraction of surface cover; and *P*_0_(*i*) is the initial *P*_0_ of the surface cover *i*. In the study area, a weighted *P*_0_ value was calculated for the entire campus to analyze the potential for surface runoff production during the campus development.

To estimate surface runoff volumes, three daily rain events (42.4, 62.4, and 78 mm), which are considered representative of the area location for return periods of two, five and ten years, respectively, were calculated by using the “Máximas Lluvias Diarias en la España Peninsular” method [52]. because of the nature of rainfall in the study area, maximum daily precipitations were considered appropriate to assess the maximum surface runoff that would be produced in extreme events. To consider the regional factor of previous soil moisture, a corrector coefficient of initial abstraction is provided by the Standard 5.2-IC to watershed calibration (Equations (10) and (11)).

(10) $$P_{0\;}\left(w\right)=P_{0}^{i}\left(w\right)\cdot \;\beta^{PM},$$

(11) $$\beta^{PM}=\beta_{m}\cdot \;F_{T}$$

where *β_m_* represents the average of the corrector coefficient of initial abstraction in the study area location (dimensionless) (*β_m_* = 2.1 in Elche); and *F_T_* is a dimensionless coefficient function of a return period *T* (for the present study: *F_T_* = 0.67, 0.86, and 1 for *T* = 2, 5, and 10 years, respectively).

#### 2.3.3. Proposed Scenarios and *P*_0_ Values Associated

The SCS-CN method was applied to three different scenarios to assess changes in runoff along the development of the campus and determine the hydrological response if better soil conservation practices had been employed. For that, impervious surfaces of roof tops were replaced by green roofs, and parking lots, roads, and pedestrian networks by permeable pavements. The aim was to establish how the surface runoff produced would differ with changes in surface cover types. The following scenarios were applied to the three daily rain events defined in the previous section (42.4, 62.4, and 78 mm):

**Scenario 1.** Conditions before campus development (1997). It is estimated that the surface is almost unaltered.

**Scenario 2.** Conditions after campus development (2017).

**Scenario 3.** Conditions after campus development (2017) with: (a) sedum roofs installed on 95% of the building area (3373.65 m^2^ of green roofs in the entire campus); and (b) permeable paving replacing the current impervious surface of parking lots, roads, and pedestrian networks (22.81 ha of the whole campus). As green roofs represented a small portion of the study area, runoff was computed considering both proposals together.

*P*_0_ values for each surface cover type were assigned as indicated in Section 2.3.2 and additional curve numbers were found in earlier research for Scenario 3. For permeable paving like porous asphalt or concrete, studies in North Carolina have shown CN values from 45 to ~85, depending on the base depth and underlying soil composition [53]; however, this study followed the recommendation of the NC Cooperative Extension of a range from 75 to 80 [54]. A CN value of 80 (*P*_0_ = 12.7 mm) was chosen for HSG C in the study area. For green roofs, many CN values have been reported in the literature in different climatic regions with considerable variations. For example, CN values ranging 93-98 for vegetated and bare green roofs were recorded in [55], while Getter et al. [20] derived CN values of 84, 87, 89, and 90 for green roofs with 2, 7, 15, and 25% slope gradient, respectively. In the present study, to ensure good drainage, a CN value of 87 for sedum roofs was established for a fixed slope of 7% [20].

## 3. Results

### 3.1. Spatiotemporal Land-Cover Changes Analysis

**Figure 2:**
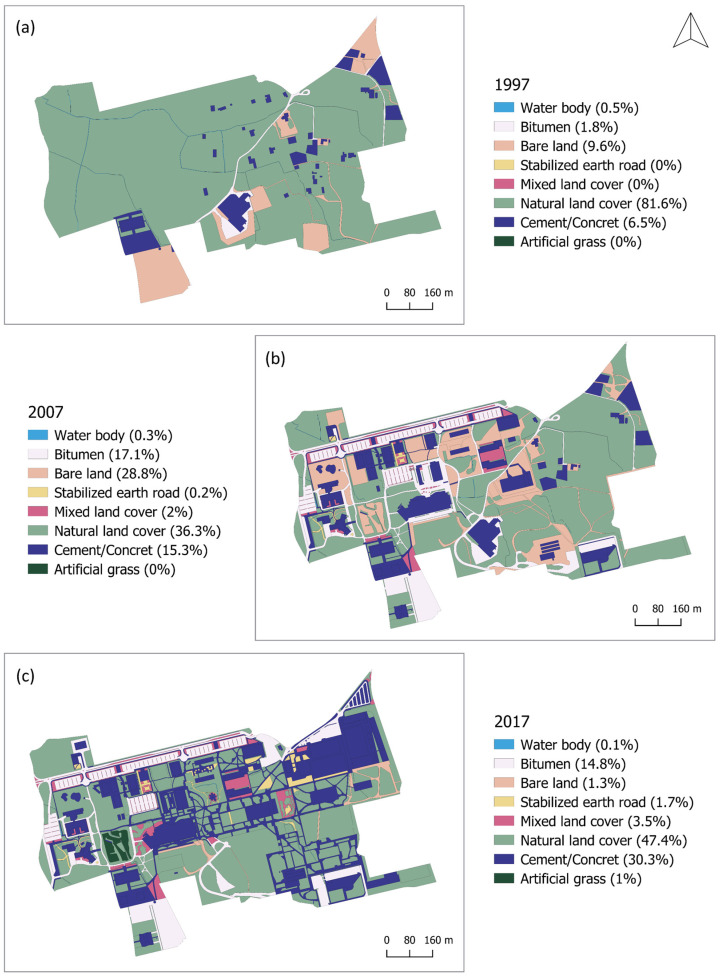
Maps of the land cover of the UMH campus in (**a**) 1997; (**b**) 2007; (**c**) 2017.

Maps from 1997, 2007, and 2017 (Figure 2) were compared to produce a cross-tabulation matrix showing the amount of surface that changed within each category. The approach of Pontius et al. [45] allowed us to answer a few increasingly detailed questions, from analyzing the net change for each category to knowing the distance at which the change occurred. In the present study, net change, gain, loss, and land swap of each category were analyzed.

**Figure 3:**
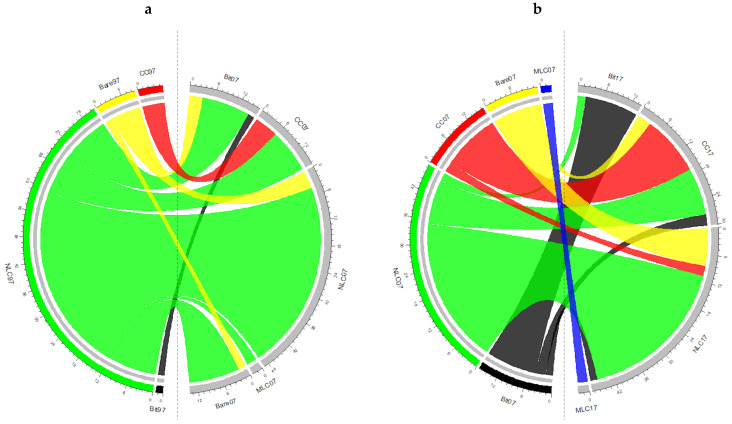
Chord diagram showing the major interrelations and changes between 1997–2007 (**a**) and 2007–2017 (**b**). Land cover abbreviations used in the diagram: Bare: bare soil; CC: cement/concrete; Bit: bitumen; ML: mixed land cover; NL: natural land cover. Years: 97 (1997), 07 (2007), 17 (2017).

Change indicators for the first (1997–2007) and second (2007–2017) periods are shown in Table 3 and in the chord diagram displaying the interrelations (Figure 3), where it can be seen that from 1997 to 2007, 63.16 ha (54.13%) of the campus surface remained constant. Hence, the total change was 31.58 ha (45.87%), of which 22.03 ha (32%) belonged to net change and 9.54 ha (13.86%) were swaps.

Natural land covers from mosaics of annual crops with permanent irrigated crops experienced the largest net loss, with an average of 21.88 ha (31.78%). On the other hand, the development of car parks, roads, pedestrian networks, and buildings resulted in major net gains to bitumen, cemented/concreted surfaces, and bare lands (14.61, 10.03, and 4.5%, respectively). Mixed land covers and earthen pavement were first introduced in small proportions into dry gardens and pedestrian networks, replacing natural covers, cemented or concreted surfaces, and irrigation channels. Throughout this first stage of the construction, it was observed that changes in bitumen, cement and concrete, stabilized earth road, and mixed land cover were nearly pure net changes; changes in bare lands from areas under construction were almost pure swap-types of change, and changes in natural land covers and water bodies consisted of both types of change. On the whole campus surface, net change was larger than change attributable to swap, and most of the change was associated with natural land covers in part because of the fact that it was the largest category in 1997 and 2007.

In the second period, a 63.86% (43.97 ha) of persistence was registered for the entire campus. Therefore, the total change was 24.88 ha (36.14%), of which 11.67 ha (16.95%) belonged to net change and 13.21 ha (19.18%) were swaps. Bare lands experienced the largest net loss, with an average of 8.83 ha (12.82%), transforming mainly into revegetated areas in urban green spaces. Least significance losses (<3%) were registered in natural covers, asphalted surfaces, and water bodies. Furthermore, a 3.72% increase in net gain was detected for cemented and concreted surfaces associated with the development of sports facilities and pedestrian networks, where natural covers, bitumen, and bare soils were transformed. Throughout this second stage, changes in stabilized earth roads, bare lands, water bodies, and artificial grass were nearly pure net changes; changes in bitumen and natural land covers were almost pure swap-types of change, and changes in cement, concrete, and mixed land covers consisted of both types of change. On the whole campus surface, the change attributable to swap was larger than the net change, and most of the changes were associated with bare lands and cemented or concreted surfaces, as they were the second largest categories in 2007 and 2017, respectively.

### 3.2. Impact of Land-Cover Changes on Surface Runoff

#### 3.2.1. Soil Sealed and Initial *P*_0_ Values in the Study Area

**Figure 4:**
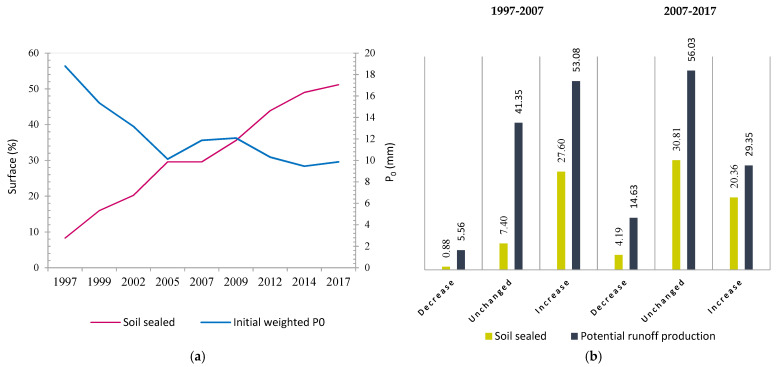
Illustration of (**a**) the evolution of soil sealing and initial weighted *P*_0_ values between 1997 and 2017; (**b**) the percent of surface that experienced changes in soil sealed and *P*_0_ values (a decrease in potential runoff production means an increase in *P*_0_ value, and vice versa).

From the chronological land-cover maps, it was possible to assess the whole campus development process and its impact on the potential for runoff production, as shown in Figure 4a. A clear difference in the proportion of soil sealed between 1997 and 2017 was observed, a period in which the campus area experienced an average annual change rate of 10.6%, until, in the last year, the soil sealed accounted for more than a half of the total area. In consequence, the initial weighted *P*_0_ value for the entire campus, set at 18.79 mm for 1997, dropped to 11.87 mm in 2007 and 9.86 mm in 2017, exhibiting a rising trend in runoff production for any precipitation event. From 1997 to 2017, *P^i^*_0_ values remained stable in 18.79% of the whole area, while 72.56% of the surface recorded a higher potential for runoff production, which was mainly associated with impervious covers.

Conversely, only a small proportion of the campus (8.65%) improved its infiltration capacity because of the removal of old buildings and asphalted roads. Figure 4b illustrates these changes for each stage of development: 1997–2007 (west sector) and 2007–2017 (east sector). Smaller increases in *P*_0_ were observed during the second period, even though the increases in sealed surfaces between both phases were similar. Replacement of cemented and concreted surfaces for less impervious covers in construction practices could explain some of these changes, for example, the use of stabilized earth pavements in pedestrian networks around the sport facilities. Besides, land use must be considered, since in the first period larger surfaces were allocated for parking and buildings, responding to a great social demand. Then, more urban green areas and dry gardens occupied the east sector.

**Figure 5:**
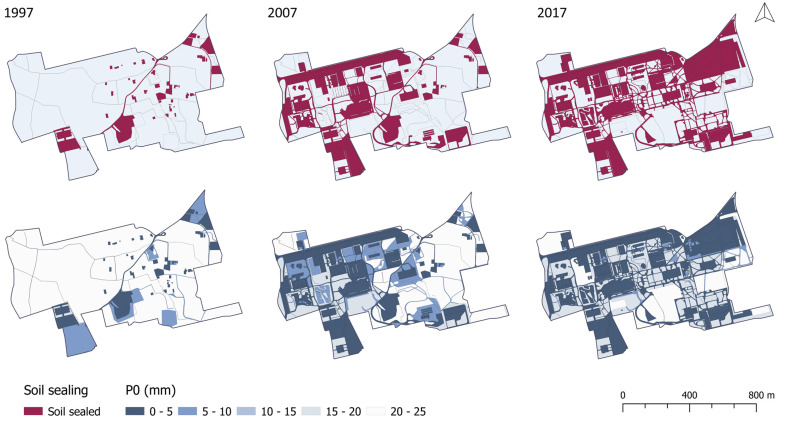
Spatial variability of soil sealed and initial *P*_0_ values between 1997 and 2017.

The spatial distribution of *P*_0_ values is shown in Figure 5, where between 1997 and 2017, a 28% reduction was detected for *P*_0_ ranging from 20 to 25 mm, and at the same time, increases of 42 and 28% were recorded in ranges of 0–5 and 15–20 mm, respectively. As a result, the study area shows a mosaic of covers, which is mainly characterized by *P^i^*_0_ values lower than 5 mm that will contribute to generate greater depths of runoff.

#### 3.2.2. Weighted *P*_0_ Values and Surface Runoff Estimation for Different Scenarios

The initial weighted *P*_0_ for modeling Scenarios 1, 2, and 3 was 18.79, 9.86, and 13.83 mm, respectively. From initial weighted *P*_0_ values, corrector coefficients were applied to each return period and runoff volumes were estimated using the SCS-CN method. As expected, for each return period, the highest runoff depth was calculated for the developed campus (Scenario 2), since the weighted *P*_0_ values were the lowest; while the runoff generated for the pre-developed situation (Scenario 1) recorded the smaller values (Table 4). Furthermore, it was found that replacing impervious surfaces on the campus (Scenario 3) reduced significantly maximum runoff volumes that could be incorporated into the rainwater drainage network. If LID practices had been implemented at the beginning of the construction process, maybe smaller dimensions for the current rainwater drainage system would have been required, which would have meant economic savings. These infrastructures act as first pollutant filters, which would have allowed an increase in potential for store and reused the exceeded infiltered water for future uses, such as garden irrigation. For the return period of 2 years, 5723.018 m^3^ was estimated for Scenario 2, while in Scenario 3, the runoff volume was 3013.430 m^3^. This means that a 47% reduction was achieved with surface cover replacing. Rather, for the 5- and 10-year return periods, the reduction was 42 and 39.5%, respectively. As a result, we determined that the difference in runoff volumes tended to decrease as the return period rose from 2 to 10 years.

## 4. Discussion

### 4.1. Impact of the UMH Campus Development on Surface Runoff Production

Land-cover changes have direct and indirect impacts on hydrological cycle. Analyzing urban surfaces as urban units with their own energy and matter exchanges can allow us to understand the effect that each surface transformation has on the water balance and anticipate future environmental issues. At that point, urban units need to be defined and classified.

The construction of the university campus arose in the context of socioeconomic transition, where energy, agricultural, livestock and fishing sectors decreased notably, giving way to powerful construction activity and a growing development of the tertiary sector [56]. This resulted in the need to provide new services like shopping centers, schools, or universities. Such transformation is manifested in the loss of high productive soils [57] and leads to a deterioration of soil ecosystem services by fragmentation of traditional agricultural landscapes, such as the agroecosystem of the Palmeral of Elche. Comparison of maps from 1997–2007 and 2007–2017 made possible the detection of the progressive increase in soil sealing in the study area, where periurban agricultural landscapes were transformed into urban landscapes. In fact, around 50% of the entire surface was identified as sealed soil in 2017, increasing from 5.7 ha in 1997 to 35.2 ha in 2017. The sealing of the campus was mainly due to the construction of buildings, parking areas, roads, and pedestrian networks, which are related to the use of impervious materials such as bitumen, concrete, and cement. However, in the second period, more earthen pavements were introduced in pedestrian networks, which indicated a change in campus management. In addition, access to vehicles was restricted when pedestrian transects replaced numerous road networks, a decision that, intentionally or not, could limit pollutant deposition on surfaces and their subsequent wash-off with stormwater runoff.

Cities development responds to specific needs and consumption patterns that are closely linked to urban water quality and quantity problems [58]. The first step to reduce water deterioration is to understand how the natural water balance is disturbed. Larger impervious surfaces decrease the infiltration rate of rainwater and reduce the soil’s capacity to hold water [59]; this intuitively leads us to deduce that greater runoff will be generated.

At the local level, variables such as soil type, morphology, and climate remain constant, and changes in hydrological response between scenarios rely on differences in land covers [60]. This study determined theoretically that, by modifying the land’s natural conditions (Scenario 1), potential runoff production increased 47.5% by decreasing initial weighted *P*_0_ values from 18.79 mm (1997) to 9.86 mm (2017). A similar proportion of soil sealed and weighted CN values have been detected in low-density residential areas [16,19]. Hydrological response depends on surface cover complex properties, but also on the duration and intensity of the precipitation [18]. The SCS-CN method cannot estimate accurate runoff values for low rainfall depths or time resolutions smaller than 24 h duration [61]. Nevertheless, a first approximation can be made by comparing two maximum daily rainfall events representatives of the area (42.4 and 62.4 mm), observing the greatest rainfall amount increases of 8.7 (1997), 35.7 (2007), and 50.3% (2017) in surface proportion where surface runoff was higher than 40 mm. This was particularly interesting, since it showed that the proportion of impervious surfaces in the campus played a key role in the runoff depth generated under higher intensity precipitations. Despite the fact that a continuous simulation approach would be a better representation for modeling scenarios under different ranges of duration and intensity of precipitation, there was a lack of information to characterize the watershed in 1997, so empirical methods were thought to be more appropriate in this case. However, we expect to use continuous models in future studies to evaluate specific soil and water conservation practices on the current campus.

**Figure 6:**
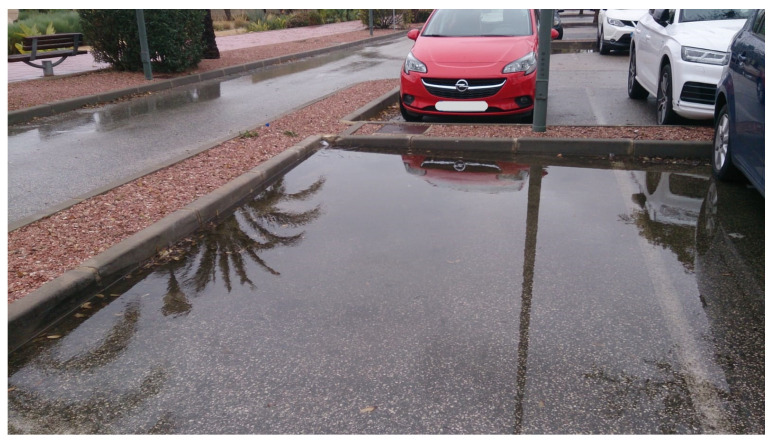
Flow accumulation in a parking lot of the study area with a total accumulated rainfall of 17.2 mm in 72 h (8 January 2021) [66].

As alternatives to mitigate surface runoff increase, nature-based solutions have been implemented around the world as a transition to more sustainable and holistic approaches to stormwater management [26]. Low Impact Development (LID) systems and practices such as bioretention cells, rain gardens, green roofs, and permeable pavements increase surface runoff volumes retention and are able to maintain or restore the original hydrologic cycle and its ecological functions in semi-arid urban areas [59,62]. As a first approximation of how the campus would have responded if green roofs and permeable pavements had been considered in the development processes, Scenario 3 was evaluated. From the results of this scenario, it was possible to confirm that replacing some impervious surfaces on the university campus could reduce the surface runoff generated in extreme precipitation events associated with 2-, 5-, and 10-year return periods by up to 40%. Such types of LID practices have not been implemented yet in the campus area; thus, some proposals and considerations were made to encourage managers to invest in future field research to move to better water and soil conservation measures. In semi-arid climates, designing practices require different considerations. For instance, harvested rainwater should be stored in closed containers because of high evaporation rates, and pollutant concentrations are larger because of the low frequency of storm events; therefore, greater capture volumes are needed for first-flush treatment [63]. On the other hand, plants should be native and drought-tolerant, but also able to tolerate inundation [63]. As examples of successful cases, permeable pavements, rain gardens, bioswales, green roofs, and bioretention ponds have been implemented in the semi-arid West of EEUU [64]. In the study area, green roofs would be a suitable practice not only because of their hydrological mitigation (reduction of peak flow and water pollution) but also because they provide insulating properties that allow reducing energy consumption related to building cooling (around 0.7%) [65], which is especially interesting in Elche, where temperatures of 38 °C are reached in summer. Despite permeable pavements helping to absorb flash floods from large storms, replacing all the conventional pavements in the campus as simulated in Scenario 3 would be extremely expensive. Nevertheless, some changes can be made by incorporating bioswales and bioretention practices instead of dry gardens or concrete, for example, around parking lot areas. Sometimes, the implementation of such practices does not necessary involve high costs, and flow accumulation as shown in Figure 6 can be avoided with simple but effective solutions, such as providing a wide opening for stormwater flow and redirecting it into garden areas [63], treating stormwater as a resource rather than a waste product.

### 4.2. Limits and Strengths

In this study visual photointerpretation of high-resolution images was carried out and eight land-cover types were identified according to the nature of their surfacing material. Some drawbacks related to work at small-size scales are linked to subjectivity in urban unit definition; firstly, due to a lack of image resolution, and secondly, because no common classifications have been established [67], resulting in different land-cover types depending on the aim of the study. Moreover, the composition of surfacing material cannot be always properly identified with aerial images, so land cover and land use have usually been employed indistinctly to refer to urban units. This work purposed to create a database that incorporated new attributes, both physical and environmental, and be used in future studies. Therefore, chronological land-cover and land-use maps were developed (Supplementary Figures S1–S3), providing detailed spatial information of urban land units integrated in the university campus, allowing different research approaches. Despite the fact that the land-cover classification mentioned in the present study was tailored to specific needs and may not be feasible for large-scale studies, it can be extended to other university campuses and improved until a common classification is created.

In the other hand, the simplicity, stability, and acceptance of the SCS-CN method make it a suitable empirical approach to modeling storm losses in the study area. Two of the greatest weakness of this lumped model are the strong dependency on a single parameter (the CN), which needs to be calibrated for each region, and the fixed initial abstraction ratio of 0.2 [68]. It is not easy to accurately select CN values to characterize a study area; for this reason, this work used both *P*_0_ and CN values from Spanish and American tabulated tables, prioritizing the first and reserving the second for those land covers that did not fit with the descriptions of the National Standard 5.2-IC. Then, a weighted *P*_0_ value was established for the entire campus and calibrated to estimate the surface runoff generated. The under- or over-estimation of runoff depth due to the weighted values of *P*_0_ increases when the CN ranges are wider [69]. For the present study, differences were considered not significant, since the purpose of this work was to compare the changes in surface runoff under different land conditions, rather than to obtain precise results. Otherwise, the initial abstraction ratio could be interpreted as a regional parameter, as values varying in the range 0–0.3 have been documented in numerous studies [70], where *λ* from 0.05 to 0.1 seems to be more representative in other locations [51,71]; consequently, additional research is needed to shed light on this issue. It should be added that hydrological responses are influenced by multiple factors and control of variables was limited to the methodology selected and data availability. It was assumed that climate conditions had an even distribution in the whole area and an average value was calculated for the entire campus, considering the morphology of the study area location and the digital elevation model used. The soil type classification remained a relevant factor and although field samples could have been analyzed for the last period (2017) to better represent soil textures in each surface cover, this process would have been too expensive and not easily done (considering permanent soil sealing). For that reason, a common soil group was defined. As a result, the most important variable between the three scenarios relied on *P*_0_ and CN values, which were considered appropriately characterized, given the available data. However, we expect to incorporate new variables in future studies to design and evaluate LID performance in the current campus conditions.

Leaving behind the limitations of this work, it is important to highlight that the flexibility of both methodologies can be adapted to other urban areas, for example, to different residential types, and can be used to evaluate the role of urban growth form in surface runoff production such as in Xu et al. [19], or we can examine the surface runoff associated with different sustainable drainage systems, given the increasing amount of experimental works that are attempting to determine CN values for the most common green infrastructures [20,55,72]. In addition, future research can be focused on investigating the surface runoff quality on the university campus, as detailed land-cover classification is already established.

## 5. Conclusions

Soil sealing is considered a parameter for measuring environmental quality, ecological footprint, and urban sustainability [73,74], so universities are faced with a planning challenge where land recycling and the integration of ecology into the design of new settlements in line with the Sustainable Development Goals will play key roles in limiting land consumption. There is no doubt that the waterproofing of surfaces has a severe impact on the hydrological cycle and, therefore, assessing and quantifying negative effects are essential to formulate comprehensive management and planning strategies that improve urban resilience to extreme climatic events, especially in semi-arid regions. By using geographical information systems and remote sensing tools in combination with the SCS-CN runoff method, this study investigated spatiotemporal land changes and estimated the surface runoff generated under three development scenarios of the university campus of Elche.

From the results it can be concluded that, first, the UMH campus follows a model of urban compact campus settlement (UCC) [35], whose proportion of impervious surface and hydrological response is similar to that of low-density residential areas. Second, by measuring the direct runoff associated with the development of the campus (Scenario 2), the impact of soil sealing as part of the urbanization process is highlighted. In general, the increment of soil sealing in the municipality of Elche due to urban growth reflects at the same time that on the university campus, although at a different scale [57]. Third, Scenario 3 confirmed the benefit of adopting LIDs as local efficient strategies, which usually can be implemented regardless of pre-existing gray infrastructures and adapted to semi-arid conditions. Furthermore, this work provides a database of chronological land-cover and land-use maps that can be extended and used in future analysis.

Among the limitations of this work, it should be mentioned that the simplification of the SCS-CN method omits other influencing factors such as intensity of precipitation or drainage systems, which should be considered in future works. Nevertheless, the flexibility of this methodology makes it an easy initiative to compare the development of other university campuses.

## Figures and Tables

**Table 1:** Descriptions of the urban land-cover classes in the study.

Land Cover	Descriptions	Images from the Campus
Bitumen	Road networks and parking areas	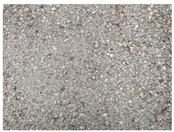
Cement/Concrete	Pedestrian networks and buildings	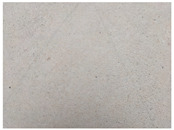
Artificial grass	Golf course	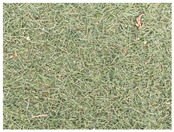
Stabilized earth road	Pavement composed of aggregates, binder, and water that is generally found in pedestrian networks	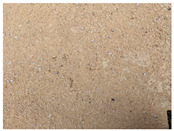
Mixed land cover	Land covered by a mix of materials that can be found in nature (gravel, sand, vegetation, wood, etc.) and artificial synthetic materials obtained by man through physical and chemical processes (e.g., geotextile membranes) associated with dry gardens	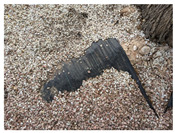
Natural land cover	Land covered by materials that can be found in nature (gravel, sand, vegetation, wood, etc.) associated with green urban areas with fully grown vegetation (including trees, shrubs, and grass) and crops	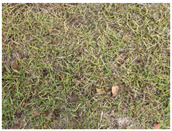
Bare land	Lands that are not covered and susceptible to erosion processes and loss of structure due to compaction, waterproofing, etc., generally land under construction, vacant lands, dirt roads, and green urban areas with grow stage vegetation	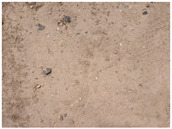
Water body	Ditches, swimming pools, ponds, reservoirs, and any other open water	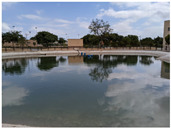

**Table 2:** Initial *P*_0_ and CN values for each land-cover type in the study area for AMC II. Equivalence to *P*_0_ for CN values after applying Equation (7) is indicated in parentheses.

Initial *P*_0_ (mm)	Initial CN	Land Cover
0		Water body
1		Bitumen, cement, and concrete
	96 (2.1)	Artificial grass, mixed land cover (dry gardens)
	87 (7.6)	Bare land (dirt road)
8		Bare land (land under construction)
	86 (8.3)	Bare land (green urban areas with grow stage vegetation: vegetation cover < 50%)
	74 (17.8)	Natural land cover (green urban areas with fully grown vegetation: vegetation cover > 75%)
22		Natural land cover (crops *)

Note: *P*_0_ and CN values were assigned according to the lookup tables of the Spanish Standard 5.2-IC and the NEH of the NRCS. * Historical crop types were obtained through the Spanish Geographic Information System of Agrarian Data (SIGA). The *P*_0_ value is associated to a “Mosaic of annual crops with permanent irrigated crops”.

**Table 3:** Land-cover changes in terms of percent of the campus surface where 1st is 1997–2007; 2nd is 2007–2017.

Land Cover	Persistence		Gain		Loss		Total Change		Net Change		Swap	
	1st	2nd	1st	2nd	1st	2nd	1st	2nd	1st	2nd	1st	2nd
Bitumen	1.67	12.04	14.71	2.76	0.11	4.34	14.82	7.09	14.61	1.58	0.21	5.51
Cement/Concrete	5.61	13.78	10.92	16.51	0.90	2.76	11.82	19.27	10.03	13.75	1.79	5.51
Stabilized earth road	0	0.36	0.36	1.29	0	0	0.36	1.29	0.36	1.29	0	0
Natural land cover	44.8	34.82	4.94	12.60	36.73	15.01	41.68	27.61	31.78	2.41	9.89	25.20
Mixed land cover	0	2.38	2.51	1.08	0	0.13	2.51	1.21	2.51	0.95	0	0.26
Bare land	1.79	0.37	12.34	0.94	7.84	13.77	20.19	14.71	4.50	12.82	15.69	1.88
Water body	0.17	0.11	0.07	0	0.29	0.13	0.35	0.13	0.22	0.13	0.13	0
Artificial grass	0	0	0	0.96	0	0	0	0.96	0	0.96	0	0
Total	54.13	63.86	45.87	36.14	45.87	36.14	45.87	36.14	32.00	16.95	13.86	19.18

**Table 4:** Weighted *P*_0_ values (*P*_0_ (*w*)) and surface runoff depth (*E*) generated on the university campus for each scenario and maximum daily precipitations of 42.4 mm (*T* = 2), 62.4 mm (*T* = 5), and 78 mm (*T* = 10).

T (Years)	Scenario 1		Scenario 2		Scenario 3	
	*P*_0_ (*w*) (mm)	*E* (mm)	*P*_0_ (*w*) (mm)	*E* (mm)	*P*_0_ (*w*) (mm)	*E* (mm)
2	26.44	1.72	13.87	8.31	19.46	4.38
5	33.93	4.09	17.81	14.88	24.98	8.63
10	39.46	6.30	20.71	20.41	29.04	12.34

## Data Availability

All the data used in this article can be accessed and are available in the following institutions: National Geographic Institute of Spain (www.ign.es, accessed on 9 May 2021 and 12 July 2021), Valencian Cartographic Institute (www.visor.gva, accessed on 12 July 2021), and U.S. Department of Agriculture, NRCS (https://www.nrcs.usda.gov, accessed on 12 July 2021); or can be provided by our university (University Miguel Hernández of Elche).

## Supplementary Materials

The following are available online at https://www.mdpi.com/article/10.3390/ijerph18189511/s1, Figure S1: Chronological Land-Cover Maps (1997–2017) of the UMH campus, Figure S2: Chronological Land-Use Maps (1997–2017) of the UMH campus, Figure S3: Chronological Soil Sealing Maps (1997–2017) of the UMH campus.
