# Supplementary material for: Soil Sealing and Hydrological Changes during the Development of the University Campus of Elche (Spain)
Source: Int J Environ Res Public Health. 2021 Sep 9;18(18):9511. doi: 10.3390/ijerph18189511 (PMC8467600; doi:10.3390/ijerph18189511)
Supplement: Supplementary file 1 [file ijerph-18-09511-s001.zip › ijerph-1372981-supplementary.pdf]

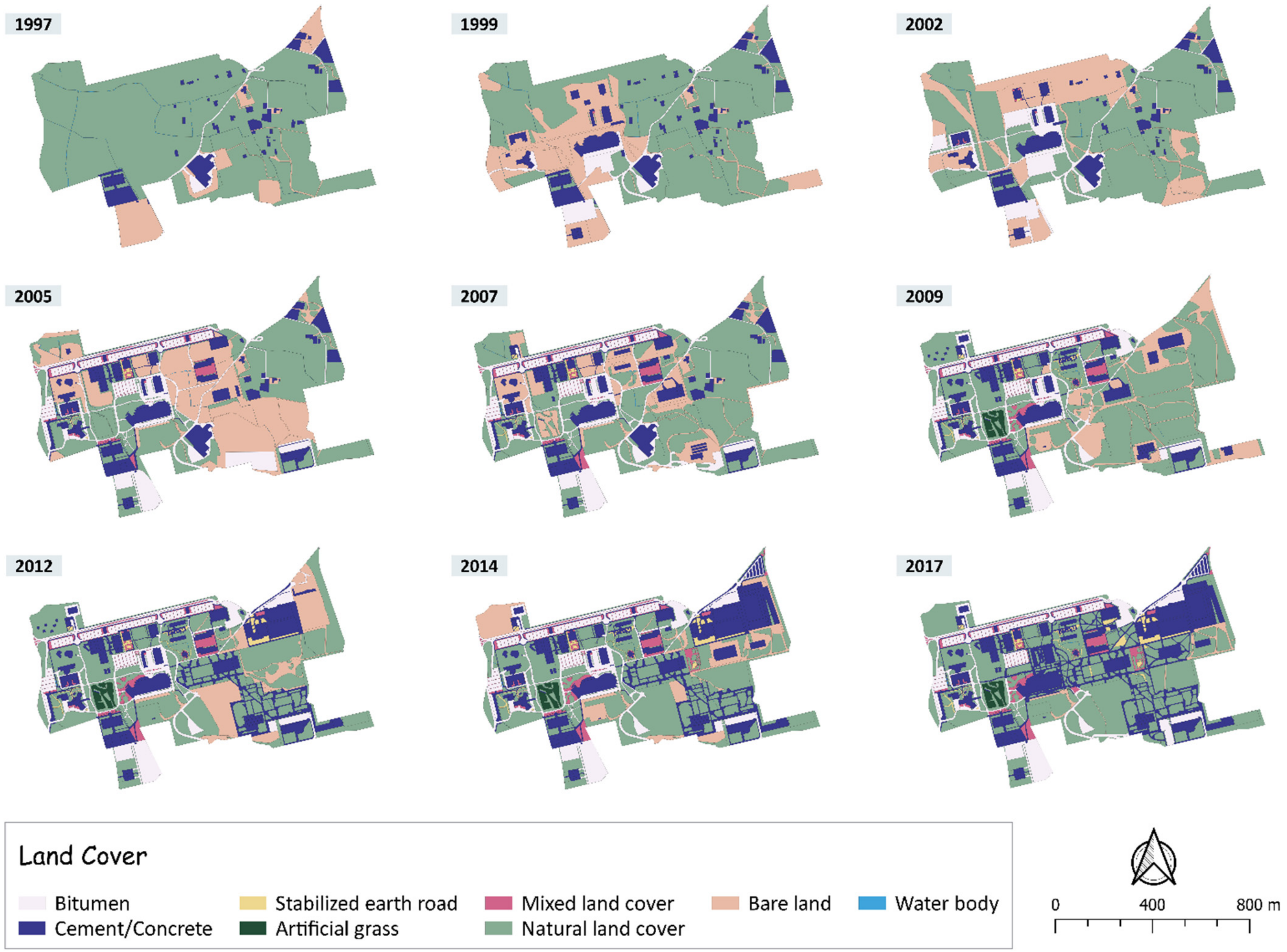

Figure S1. Chronological Land Cover Maps (1997-2017) of the UMH campus.

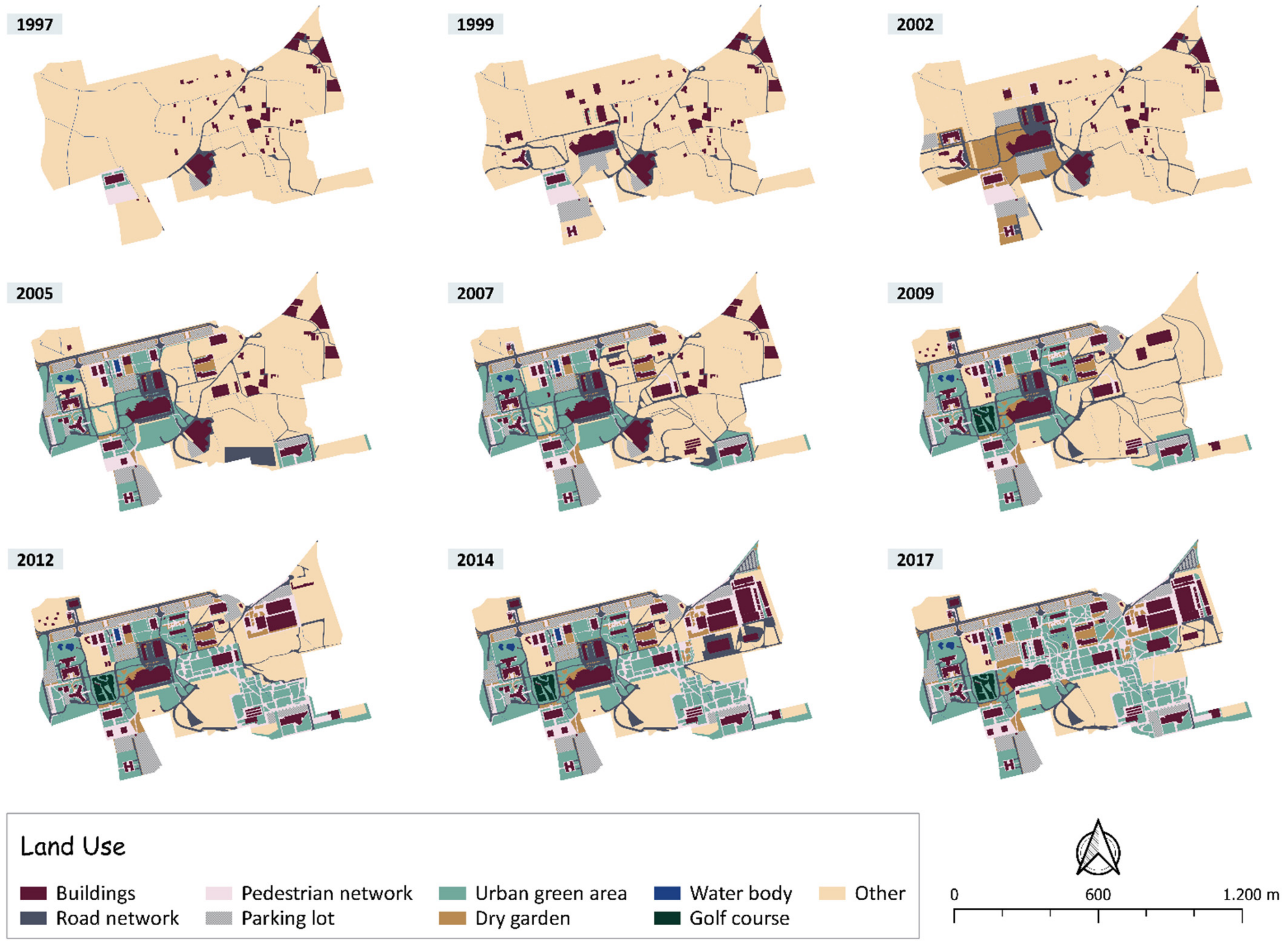

Figure S2. Chronological Land Use Maps (1997-2017) of the UMH campus.

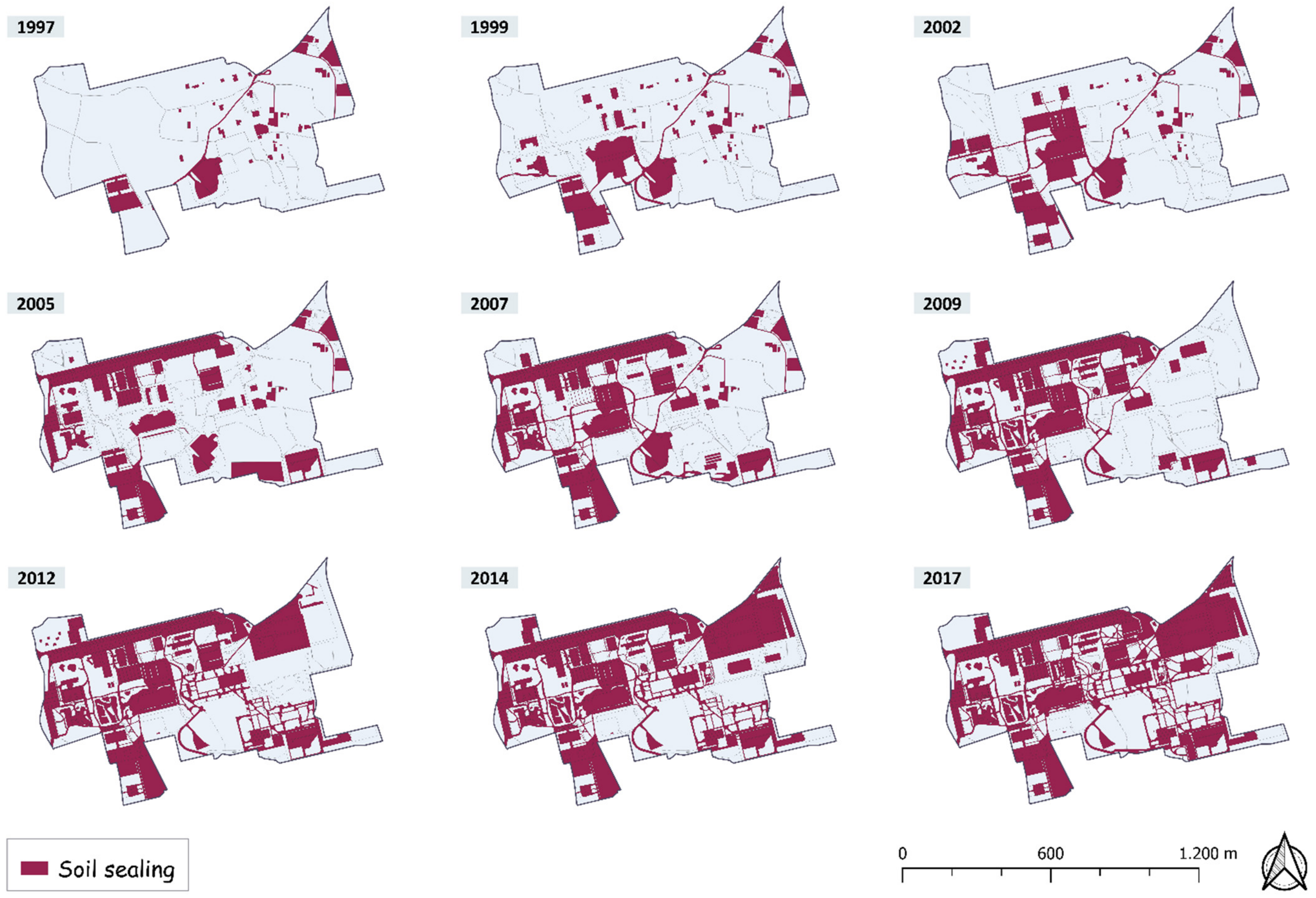

Figure S3. Chronological Soil Sealing Maps (1997-2017) of the UMH campus.
